# Stress and coping – Perceptions of final year nursing students returning to clinical practice during the COVID-19 pandemic, South Africa

**DOI:** 10.4102/hsag.v26i0.1641

**Published:** 2021-11-05

**Authors:** Mary Ann Jarvis, Penelope Martin, Margaret Williams, Fiona Walters, Olivia B. Baloyi, Jeffrey Hoffman, Jennifer Chipps

**Affiliations:** 1 Discipline of Nursing, College of Health Sciences, University of KwaZulu-Natal, Durban, South Africa; 2 School of Nursing, Faculty of Community and Health Sciences, University of the Western Cape, Cape Town, South Africa; 3 Discipline of Pharmaceutical Sciences, College of Health Sciences, University of KwaZulu-Natal, Durban, South Africa

**Keywords:** clinical practice, coping, COVID-19 pandemic, nursing students, stress

## Abstract

**Background:**

The novel nature of the coronavirus disease 2019 (COVID-19) pandemic places challenges on nursing students as they try to complete the clinical requirement of their training. Nursing faculties need to understand these challenges to support and equip nursing students to enter the workforce.

**Aim:**

To explore and describe the anticipated and subsequent perceptions of final year Bachelor of Nursing students returning to clinical practice during the COVID-19 pandemic in South Africa.

**Setting:**

The study was conducted at two universities in the Western Cape and KwaZulu-Natal, South Africa. Both universities offer 4-year Bachelor of Nursing programmes accredited by the South African Nursing Council and were in ‘hot spot areas’ for SARS-CoV-2.

**Methods:**

A qualitative study with focus groups discussions of final year undergraduate nursing students from both universities were conducted. Data were analysed through content analysis using Lazarus and Folkman’s Stress Appraisal Coping Model.

**Results:**

Five focus groups discussions with a total of 25 participants were conducted. Three themes with eight sub-themes emerged, the key themes being: primary appraisal and anticipation of returning to clinical practice; contextual influence on primary appraisal and reappraisal to facilitate positive return to clinical practice.

**Conclusion:**

Primary perceptions of returning to clinical practice revolved around uncertainty and stress. However, through preparation and the process of reappraisal, participants were able to adapt and cope with the challenges in returning to clinical practice during the pandemic.

**Contribution:**

It is important to recognise the role of faculty in supporting nursing students’ transition into situations of uncertainty such as the pandemic.

## Introduction

Globally, the ongoing coronavirus disease 2019 (COVID-19) pandemic has been a stressful period with the need to adapt to constantly emerging new information (CDC 2020). Healthcare workers, specifically nurses and nursing students, have been on the frontline, caring for COVID-19 patients and witnessing colleagues, family, friends and patients succumb to the virus (Casafont et al. 2021). COVID-19 has resulted in an increased workload in healthcare facilities combined with concerns about personal protective equipment (PPE), fears of self or family being exposed to the virus and concerns about financial and social support (Kackin et al. 2020). There is also the additional concern of transmission between these settings for nursing students, with a direct link between hospitals and universities (Di Gennaro et al. 2020).

Research published during the pandemic has primarily focused on anxiety and fears experienced by nurses in practice (Despoina & Chrysoula 2020). While not yet qualified, nursing students have also been rendering care to patients infected with SARS-CoV-2 and faced with rapid changes in the clinical settings (Taylor et al. 2020). Though infection prevention and control are core parts of nurse training (Abdelaziz, Dogham & Elcokany 2019), the novel nature of the pandemic, the high level of transmissibility of the virus (Di Gennaro et al. 2020) and the move to online teaching and learning (SA Government 2020) have resulted in additional challenges for nursing students already under pressure to complete their degrees. This has resulted in increased anxiety impacting academic functioning (Cao et al. 2020). In South Africa, nursing students were provided the option not to return to clinical practice at this time; however, most nursing students opted to return to complete their qualification. International reports indicated that nursing students who decided to return to practice expressed fears of becoming infected (Gómez-Ibáñez et al. 2020).

In this new situation, filled with uncertainty (Anicich et al. 2020), fear (Despoina & Chrysoula 2020) and a loss of control, there is a higher sense of risk to health workers (Human Sciences Research Council [HSRC] 2020). In this environment, the nursing faculty is responsible for ensuring that structures are put in place to facilitate coping in the changed clinical setting (Cao et al. 2020; Nelson & Lee-Winn 2020; Townsend 2020). Understanding nursing students’ anticipated perceptions of the pandemic should enable nursing faculty to support and prepare students for work during a pandemic. This would allow them to develop self-agency by utilising adaptive coping mechanisms following appraisal of their environment and a sense of control over their actions.

## Aim

The aim of the study was to explore and describe the perceptions of final year Bachelor of Nursing students’ return to clinical practice during the COVID-19 pandemic in South Africa.

### Definition of key concepts

**Stress** is defined as a highly individualised response to a stressor (Lazarus & Folkman 1984). In this study, stress is the students’ subjective response to the stressors within the clinical environment during the COVID-19 pandemic.

**Coping** is ‘the constantly changing cognitive and behavioural efforts to manage the specific external or internal demands that are appraised as taxing or exceeding the resources of the person’ (Lazarus & Folkman 1984). Coping in this study refers to students’ cognitive and behavioural efforts when they evaluated encounters in the clinical placement setting as being stressful.

## Research method and design

### Study approach and design

A qualitative, exploratory, descriptive (Creswell 2013), contextual study was conducted using qualitative online focus group discussions to allow for broad-based questions and the emergence of new information unique to the pandemic.

### Study setting

The study was conducted at two universities in two of the nine provinces in South Africa located in ‘hot spot areas’’ for SARS-CoV-2 (Abdool Karim 2020). University A is in the Western Cape, home to approximately 7 million people (Statistics SA 2020). University B is in KwaZulu-Natal, on the east coast, with an estimated 11.5 million people (Statistics SA 2020). Many of the students from both these universities were from under-resourced and previously disadvantaged areas.

Both universities offer 4-year Bachelor of Nursing degrees, accredited by the regulatory body the South African Nursing Council (SANC), requiring 4000 h of clinical practice (SANC R.425 programme). During the initial full lockdown (Level 5) in South Africa starting in March 2020, all academic activities migrated online, residences were closed and students returned to their primary residences. In May 2020, final year students were allowed to return to campus and clinical settings to complete their degree as part of Level 4 lockdown (SA Government 2020). As there were no changes to the required 4000 h for the BN (R.425) programmes (SANC 2020), the universities commenced academic activities with an orientation to return the final year nursing students by the end of July 2020 to complete the outstanding requirements in clinical hours. The universities collaborated and developed a compulsory online orientation to SARS-CoV-2 and preparation to return to the clinical setting. This orientation included information on SARS-CoV-2, a revision of infection control principles, strategies to deal with anxiety and ongoing digital support using WhatsApp groups.

### Study population and sampling strategy

The population for the study was the final year nursing students from the two universities. Purposive sampling strategy was used to select all students in the final year of the nursing programme (*n* = 266), from University A (*n* = 198) and University B (*n* = 68) (Suri 2011). All final year students were invited through the WhatsApp support groups to participate in online focus group discussions. Eligibility criteria included all fourth-year nursing students who were able to access the online discussion forum and were willing to participate in the focus group discussions.

### Data collection

Five online focus group discussions were conducted from 09 to 23 September 2020, using a formal focus group arrangement (Creswell 2013). Focus group discussions conducted by four of the researchers who are all PhD graduates, experienced in conducting group discussions, were known to the students as they had taught a module to the students in the fourth year. The interviewers were familiar with the universities’ practices in the return of the students to clinical practice. The focus group discussions were audio-recorded and lasted between 45 and 90 min. Two focus group discussions were conducted at University A and three at University B, with each group having a facilitator and moderator, managing interactions between the participants (Taylor, Bogdan & De Vault 2015). The facilitator posed the questions and probes to the students, and the moderator sought further clarification to gain insight into the students’ experiences and where aspects needed further probing. Data collection continued until data saturation occurred after the fifth focus group discussion yielded no new data (Hennink, Kaiser & Weber 2019). A focus group guide was used to collect the data, using one open-ended question with probes: *What were your experiences as a nursing student in returning to clinical practice during the COVID-19 pandemic*?

### Data analysis

Lazarus and Folkman’s (1984) stress-appraisal-coping model, focusing on the interaction between an individual and the environment as a framework, and Tesch’s open coding method as outlined by Creswell (2013), was used to analyse the data. The collected data were transcribed verbatim, followed by member checking. All transcripts were read and re-read to make sense of the whole by five of the researchers. Four researchers coded manually, while one researcher used Atlas.Ti, version 8.4.3 qualitative data analysis software package. Themes were created from the identified sub-themes, and anecdotal notes were used to assist during the latter process. The three concepts, primary appraisal, transaction with the environment and reappraisal from Lazarus and Folkman’s Stress Appraisal Coping model (Lazarus & Folkman 1984) guided the construction of the themes.

### Trustworthiness

The four measures of confirmability, transferability, dependability and credibility ensured trustworthiness (Creswell 2013; Shenton 2004). To ensure confirmability in the study, all researchers analysed the transcripts individually (Creswell 2013). The researchers then met to compare and discuss the sub-themes and themes to reach a consensus (Creswell 2013). Rich, thick descriptions of the setting and participants’ demographics were given to allow decisions around the transferability of the findings (Creswell 2013). All interviewers are experts in qualitative research, which provided dependability and credibility to data collection (Shenton 2004). Furthermore, credibility was ensured through the audit trail, which included audio-recording and verbatim transcription of the focus group discussions and critical review by a research team member not involved in group discussions (Shenton 2004).

### Ethical considerations

In upholding the ethical standards of conducting qualitative research, this study adhered to the principles of respect for human dignity, beneficence, privacy and confidentiality (Vanclay, Baines, & Taylor 2013). In ensuring beneficence, the research ethics committee (information redacted to maintain the integrity of the review process) provided ethical approval. Moreover, permission for student participation in the study was obtained from the registrars of both universities (Vanclay et al. 2013). Participants volunteered to participate in the online focus group discussions (Sugiura, Wiles & Pope 2017). Before the focus group discussions commenced, the participants verbally consented to participate and allowed audio-recording of the focus group discussions for data analysis (Vanclay et al. 2013). They also typed their consent in the comment chatbox (Vanclay et al. 2013). The interviewers allowed the participants to select preferred pseudonyms to ensure confidentiality (Vanclay et al. 2013). All participants were treated fairly and provided with information before participation through an information sheet, which stipulated the purpose of the research, duration of participation and the voluntary nature of participation, information remaining within the confines of the group, coupled with the opportunity to withdraw before the audio-recording began, without any adverse repercussions (Vanclay et al. 2013).

Permission to conduct the study: The researchers wrote letters to the specific authorities requesting permission to undertake the study at their respective institutions. The purpose of the study, the target population and how the participants were selected and their involvement throughout the study are clearly stated in the letters. Further explanations were provided to the authorities regarding ethical considerations of the study. The researchers were available to respond to any queries or concerns if there were any from the authorities. Ethics approval was obtained from the Humanities and Social Sciences Ethics Committee (HSSREC) of the University of the Western Cape (reference number: HS20/7/3) and gatekeeper permission was granted by the University of the KwaZulu-Natal.

Informed consent: Through the Learning Management System of both universities, the researchers provided the participants with information letters, two days to internalise the contents of the letter. Thereafter, the researchers obtained informed consent in electronic format from all the participants.

Voluntary participation: The researchers informed the participants that their participation in the study was entirely voluntary and that they may withdraw from the study at any given time without any penalty. The researchers are academic staff at the participating universities, and hence the students were reminded that their participation was anonymous and non-participation would not influence their grades or academic performance in any manner.

Risk of harm: As with all research, there is always a risk of harm to participants. In this study, the researchers were aware that through data collection the participants may have become more acutely aware of their anxiety towards COVID-19. Student support services details were provided for participants who may have wished to access these services on an individual basis. Arrangements for access to the student support services of both universities were made prior to conducting the study.

Design choice: A one-group comparison versus a randomised control study was selected in light of the severity of the virus and the opportunity for the student to mitigate the anxiety and correctly provide for personal safety, including that of the patient.

Statement of confidentiality and anonymity: The researcher ensured the confidentiality and anonymity of the participants. All data collection devices, that is, audio-recordings and transcripts were password protected and known only to the researchers.

## Results

*Participants:* A total of 25 participants consented to participate in the study (Table 1). Participants included 12 male and 13 female final-year nursing students. The mean age of the participants was 22.8 years (range 21–29 years), and just over half (*n* = 13, 52%) was from the rural areas (Table 1)

**TABLE 1:** Demographic profile of study participants.

Participant	Sex	Age	Primary residence
FGA1: P1	Male	25	Rural
FGA1: P2	Male	21	Rural
FGA1: P3	Female	22	Urban
FGA2: P4	Female	23	Urban
FGA2: P5	Male	25	Rural
FGA2: P6	Female	29	Urban
FGA2: P7	Male	24	Urban
FGA2: P8	Female	21	Rural
FGA2: P9	Male	23	Rural
FGB1: P10	Female	21	Urban
FGB1: P11	Female	22	Rural
FGB1: P12	Male	22	Urban
FGB1: P13	Male	21	Urban
FGB1: P14	Male	24	Rural
FGB1: P15	Female	24	Urban
FGB2: P16	Male	22	Rural
FGB2: P17	Female	21	Urban
FGB2: P18	Male	21	Rural
FGB2: P19	Male	22	Rural
FGB2: P20	Male	27	Rural
FGB3: P21	Female	21	Urban
FGB3: P22	Female	23	Rural
FGB3: P23	Female	21	Urban
FGB3: P24	Female	21	Urban
FGB3: P25	Female	25	Rural

FGA: Focus group University A; FGB: Focus group University B.

*Themes and sub-themes*: Aligned with Lazarus and Folkman’s Stress Appraisal Coping model (Lazarus & Folkman 1984), three themes with eight sub-themes emerged, namely primary appraisal and anticipation of returning to clinical practice; contextual influence on primary appraisal and reappraisal to facilitate a successful return to clinical practice (Table 2).

**TABLE 2:** Themes and sub-themes.

Themes	Sub-themes
Primary appraisal and anticipation of returning to clinical practice	1.1 Uncertainty about the novel, unknown experience
	1.2 Perceptions of fear related to the clinical setting
	1.3. Mixed feelings due to anticipation of the completion of degree
Contextual influence on primary appraisal	2.1 Concern and support for families during the pandemic
	2.2 Cultural and geographical influences on perceptions
	2.3 Media’s influence on perceptions
Reappraisal to facilitate positive return to clinical practice	3.1 The positive effect of the orientation and support by faculty
	3.2 Developing adaptive coping strategies

Although the focus was about returning to clinical practice, the students also included their perceptions about returning to the university during the COVID-19 pandemic, describing uncertainty and mixed feelings. They had thoughts of uncertainty about what to expect in the clinical settings, experienced feelings of stress, fear and anxiety and expressed concerns about PPE and safety for themselves and their families. This was alongside positive thoughts about the possibility of completing their degrees. The transactions with their environments reflected the influence of the specific contexts, that is their families, geographical location, culture and how the media portrayed COVID-19. However, through the faculty-provided orientation, online support and the actual process of returning, the participants started to reappraise and re-evaluate the situation, showing adaptive coping strategies.

### Theme 1: Primary appraisal and anticipation of returning to clinical practice

Initially, in anticipation of returning to clinical practice, the participants’ primary appraisal was stress, anxiety and fear. Under Theme 1, three sub-themes emerged, namely uncertainty about the novel, unknown experience, perceptions of fear related to the clinical setting and thirdly, mixed feelings due to the anticipation of the completion of their degree.

#### Sub-theme 1.1 Uncertainty about the novel, unknown experience

The participants’ primary appraisal of returning to clinical practice was described as thoughts of being uncertain about this new unknown threat and having inadequate knowledge about SARS-CoV-2. The following direct quotations support the uncertainty and worry experienced by the participants:

‘I didn’t even know what was gonna happen…’ (FGA2:P6, female, aged 29)

‘…uncertainty was still there…’ (FGA2:P9, male, aged 23)

‘…because in my mind COVID was or is everywhere…’ (FGA1:P1, male, aged 25)

Although the students received PPE, their reflections of the process illuminated their fears and concerns, which centred around its timeous arrival, to ensure them working in the practice setting during the pandemic. The direct quotations below illustrate the participants’ experience of their fears and concerns:

‘…you guys gave us enough PPE. You gave us right the PPE…’ (FGA1:P3, female, aged 22)

‘I think that because of the delay in receiving PPE…I was struggling.’ (FGA2:P8, female, aged 21)

‘…the initial batch of scrubs, PPE, were given to students a week after the agreed date.’ (FGA2:P5, male, aged 25)

‘…most concerning thing that we had was the issue of PPEs …, so that thing was causing anxiety and stress.’ (FGB1:P14, male, aged 24)

‘We’re going to clinical practice but we’re more exposed and we have been exposed by the Department …it was the responsibility of the University to provide PPE’s for us just like that promise even to the South Africa because they publish the thing saying they will provide PPE’s for student.’ (FGB1:P14, male, aged 24)

#### Sub-theme 1.2 Perceptions of fear related to the clinical setting

The participants expressed perceptions of fear and anxiety related to the clinical settings. The clinical setting was now perceived as dangerous due to fears about being infected. In some cases, the anxiety was expressed through physiological responses such as palpitations and nausea. The following direct quotations support the participants’ experience of fear:

‘…shivering at some point, getting hot, even my gut when I think about going to clinical areas, I was anxious.’ (FGB2:P16, male, aged 22)

‘I had elevated anxiety and fear of exposure to COVID. My mental health deteriorated. I was very scared to return to campus because I knew I would be exposed to my friends and colleagues, so I was very sad and scared.’ (FGB3:P22, male, aged 23)

‘…affected physically like I was feeling nauseous, it just made me very nervous…have this feeling in my hands…like I couldn’t use my hands, I would get palpitations as well sometimes…’ (FGB1:P10, female, aged 21)

‘I think I was quite scared because of everything the media portrayed, so I was scared about going back into placement.’ (FGA2:P8, male, aged 21)

‘scared at first, because obviously we didn’t know what was the severity of COVID…’ (FGA2:P7, male, aged 24)

‘…staff members at the facilities were passing away or contracting the virus. I think that kind of like makes us more you know, agitated towards the whole pandemic…’ (FGA2:P9, male, aged 23)

‘I was also nervous prior to clinicals because we didn’t know what to expect at work…lot of information….a lot of people infected the nurses and they were dying …’ (FGA2:P6, female, aged 29)

‘My fear was more about infecting other people than worrying about my wellbeing.’ (FGB3:P23, male, aged 21)

The participants of one university also expressed a sense of powerlessness against what was perceived as the university’s decisions about their return to the clinical setting. The following direct quotations support the participant’s experience of powerlessness:

‘…felt like they were putting my life in danger while I am still young.’ (FGB1:P13, male, aged 21)

‘It felt like that the university is gambling with our lives…’ (FGB1:P12, male, aged 22)

#### Sub-theme 1.3 Mixed feelings due to anticipation of the completion of degree

For the participants, a major sub-theme that emerged was how the pandemic was impacting their future profession. This was accentuated by mixed feelings about completing their degree against the backdrop of the pandemic. The following direct quotations support the participants’ experience of having mixed feelings about the pandemic:

‘Yes I had mixed feelings I was anxious but at the same time I was ready to go back [*to the clinical facilities*].’ (FGB1:P15, female, aged 24)

‘I hadn’t been in the clinical areas for a long time….I was happy to be starting again because I was very stressed out about completing my degree before that…’ (FGB3:P24, female, aged 21)

‘I felt a bit nervous but I changed my mind …my family told me that I can’t run from COVID-19 and I chose this profession….I train my mind…..I choose nursing so along the line I will come across with another pandemics so I have to be strong as much as I am a little bit anxious….I was so ready to go to the clinical facilities …’ (FGB1:P11, female, aged 22)

‘I think I was quite scared …going back into placement and contracting COVID …but I was also willing to work for the hours…’ (FGA2:P15, female, aged 21)

### Theme 2: Contextual influence on primary appraisal

The transaction with the participants’ primary residence and environments before returning to clinical placement was reported as influencing their cognitive and emotional responses to the pandemic. This included the different influence of families, cultures, geographical locations and the general role of the media, emerging as three sub-themes influencing the participants’ primary appraisals of return to the clinical settings.

#### Sub-theme 2.1 Concern and support for families during the pandemic

The participants’ families played a significant role in how the participants anticipated their return to the clinical settings during the COVID-19 pandemic. These feelings included concern that their family members had for them in the face of the pandemic and their concern about not infecting their family when they returned from the clinical setting. The following direct quotations support the participants’ experiences of concern for their families:

‘I was scared that maybe I will…contact COVID-19 and will take it back to my family.’ (FGB1:P13, male, aged 21)

‘I was frightened that if I did contact it I will definitely bring it back to my family and it wasn’t more about me, it was about my family because I live at home so, they would all get it.’ (FGB1:P15, female, aged 24)

‘Contracting COVID and then bringing it back to my family…the general feeling that I had was probably scared [*of infecting family members*].’ (FGA2:P8, female, aged 21)

‘I wasn’t really worried about contracting the virus itself. It’s more the effects it would have on my family as a result of me infecting them.’ (FGA2:P15, female, aged 29)

‘I live with my family and 2 of my family members suffer from chronic disease, so I was more scared of infecting them more than me getting infected.’ (FGA2:P6, female, aged 29)

‘No I didn’t think that I will die, but I was nervous that maybe I will infect others, and then they will get sick and die.’ (FGB3:P25, female, aged 25)

‘If I develop COVID and I passed it on to him [brother] he wouldn’t be able to complete his matric year properly…I’m still not worried about contracting the virus itself…more the effects it would have on my family…’ (FGA1:P15, female, aged 22)

Similarly, family members were also reported as concerned about the participants contracting the virus. The following direct quotations allude to the experience of participants about their families’ concerns for them:

‘No they [*family*] are just scared for my life because my home is so far from the residence where I stay.’ (FGB1:P12, male, aged 22)

‘My mother [*was*] worrying about me coming back to Durban…considered a high spot for COVID.’ (FGB2:P18, male, aged 21)

‘This felt scary [*to return to campus*]… I felt anxious and my family too is [*at*] home [*rural area*]…they told me to stay indoors…[*but*] I was knowing for sure I’ll be at clinical placement.’ (FGB1:P12, male, aged 22)

‘…even though I was also anxious about COVID-19, but my worry about my mother worrying about me was more than my anxiousness about COVID-19. I was more worried about her worrying about me.’ (FGB2:P18, male, aged 21)

‘…even though I was worried about other certain things, but above it all, my biggest worry was about my mother worrying about me.’ (FGB2:P19, male, aged 22)

Most families were supportive of the participants and shared their concern that the participants were ‘out there’ away from them and their protection. The following direct quotations support the participants’ experience of their families’ support:

‘Family they were just, they are supportive.’ (FGB1:P15, female, aged 24)

‘Family calling me frequently telling me that my life is more valuable than a degree….go back home where I will be safe.’ (FGB2:P16, male, aged 22)

Families also offered encouragement in the face of the pandemic as alluded to by the following direct quotation by a participant of the experience of encouragement:

‘My family told me that maybe along the line, when I will be a professional someday, another pandemic will come. I have to face it…’ (FGB1:P11, female, aged 22)

For some participants, the family was concerned about the risk they posed to themselves and others within the family structure if they were to return home after being in the clinical settings, resulting in participants being discouraged from returning home to visit. The following direct quotations support the participants’ experience of being discouraged from returning home:

‘The [*parents*] didn’t want me to stay at home…they wouldn’t be able to see their parents so I had to go and stay by myself…’ (FGB1:P10, female, aged 21)

‘[*family*] is concerned that I would go back to res, and when I miss home, I will come back with the virus and infect them.’ (FGB3:P22, female, aged 23)

#### Sub-theme 2.2 Cultural and geographical influences on perceptions

The participants’ micro-family cultures and macro-ethnic cultures also contributed to how they perceived the pandemic. Some participants indicated that their families or communities did not believe that COVID-19 was real and believed that traditional medication would be effective to treat the virus. The following direct quotations support the participants’ experience of the cultural influences on the pandemic:

‘Like kind of prepared my mind to go back and plus with my family some of them don’t even believe about COVID, they don’t believe it’s real, so every time I saided [*said*] to tell my mum, like I’m scared, she would be like nah, you will be fine nothing will happen to you and stuff.’ (FGB1:P15, female, aged 24)

‘…ahh for me um as I stay in the rural areas, um people in the rural areas were not taking precautionary measures….was those people who were saying that they won’t get COVID-19 they are Zulu enough, they will just clean there they will just clean themselves they will just they will just take their traditional medicine and this Chinese thing can’t defeat them.’ (FGB1:P13, male, aged 21)

The need to return from the rural to the urban areas where the universities and the clinical settings were located resulted in some concern for the students and their families as they feared the urban areas, perceived to be the source of SARS-CoV-2. The following direct quotations support the participants’ experience of their families and their own concern:

‘My mother was so worried about me getting infected…when I came to Durban [*from where participant stayed in rural area*] I got more anxious because no one was wearing a mask.’ (FGB2:P20, male, aged 27)

‘I felt safe at home because I’m coming from the rural areas…when I heard that I had to come back, I was a bit nervous …’ (FGB3:P25, female, aged 25)

‘I come from a small town…people [*in Durban*] did make me more nervous because there are bigger crowds…makes it scarier.’ (FGB3:P24, female, aged 21)

#### Sub-theme 2.3: Media’s influence on perceptions

Several participants highlighted the role the media had in influencing their perceptions. These participants perceived the media reports as increasing their anxiety and fear. The direct quotations support the participants’ experience of the role the media played during the pandemic:

‘A lot of information going on, on social media about people…nurses dying…kind of nervous.’ (FGA1:P3, female, aged 22)

‘I think I was quite scared because of everything the media portrayed, so I was scared about going back into placement.’ (FGA1:P8, female, aged 21)

‘What made us more anxious is the media; people were busy posting, and you couldn’t tell the reliable sources and people who were posting fake news…Durban more deaths than Gauteng. University students are more dead than other universities, made us more worried…’ (FGB2:P19, male, aged 21)

One participant alluded to COVID-19 being exaggerated as worse than what it actually was. The following direct quotation supports the participants’ experience of the exaggeration of the pandemic:

‘Being strictly honest about it…I thought that COVID had been blown up to a degree…I just actually thought everyone was being a bit of a drama Queen…’ (FGA1:P3, female, aged 23)

### Theme 3: Reappraisal to facilitate positive return to clinical practice

The third theme that emerged was the reappraisal process that facilitated a positive return to clinical practice. During the focus group interviews, many participants differentiated between their initial appraisal and reappraisal after orientation and actual return to clinical practice. This reappraisal process helped them address their initial thoughts of uncertainty and perceptions of fear and move towards a more positive attitude towards returning to the clinical setting. According to Lazarus’ model (Lazarus & Folkman 1984), this reflection improved the participants’ internal locus of control and coping and illustrated a move towards self-agency. Two sub-themes were identified, namely the positive effect of faculty’s orientation and support and developing adaptive coping strategies.

#### Sub-theme 3.1 The positive effect of the orientation and support by faculty

A sub-theme that emerged was the importance of students’ orientation and preparation to return to clinical practice. The online support and interaction with lecturers and clinical instructors before returning to clinical practice were a positive experience. The following direct quotations support the participants’ experience of support from faculty:

‘We really had to read to fully understand what COVID was, how it transferred from one person to another, and how to prevent the spread of it.’ (FGB2:P20, male, aged 27)

‘I was scared but then as time goes now I’m okay because all the necessary counselling were done to me, so now I know how to look after myself a little bit better than before.’ (FGB1:P13, male, aged 21)

‘Information I get from you [*facilitators*] prepared me to go to the hospital, so it helped me a lot.’ (FGB1:P11, female, aged 22)

‘The WHO courses … just to reiterate that it’s very important to use PPE, it did help with my stress and anxiety…I was prepared to go back.’ (FGB1:P10, female, aged 21)

‘The digital platforms did help, answering the COVID Pack [*course*] there is a lot of information that we obtained there and it gave us more clarity.’ (FGA1:P3, female, aged 22)

Participants also expressed how the information they received to deal with the pandemic lessened their anxiety. The following direct quotations support the participants’ experience of informational support:

‘It’s not easy for me to get infected now because online information helped me in that way.’ (FGB2:P16, male, aged 22)

‘I told my family and friends that they need to wear a mask to protect themselves and others.’ (FGB2:P19, male, aged 22)

‘Effective [*online course work*]…reduced anxiety a little…’ (FGA2:P8, female, aged 21)

While anxiety persisted, the orientation enabled participants to be more optimistic about their return to a different clinical practice. The following direct quotations support the participants’ experience of being positive:

‘I was so ready to go to the clinic facilities …I was so ready, and I’m still ready.’ (FGB1:P11, female, aged 22)

‘The studies opened my mind about the virus it also lowered my fear about COVID-19’. (FGB3:P25, female, aged 25)

‘Very helpful [*online learning*]…it gave me some hope that I could manage the situation and try the best to prevent infection. Also, it was a dark time for me, so I saw the light in the tunnel.’ (FGB3:P22, female, aged 21)

‘Assurance that we are actually getting some information or backing up from the [*university*] …where we were unsure but it brought reassurance to us if I can say …’ (FGA2:P9, male, aged 23)

Some of the participants were relieved to go back to clinical practice without the concern of infecting their family members and others reported that this process provided an opportunity to educate their families on how to stay safe by engaging with universal precautions in infection control. The following direct quotations support the participants’ experience of educating their families on safety precautions:

‘I was happy that I wouldn’t be staying with my parents because I have elderly, sickly parents, so I was happy that I wouldn’t be with them. I am kind of isolated here in Durban…I was a bit more relieved.’ (FGB3:P24, female, aged 21)

‘After the [*online*] sessions I was more cautious…limited my contact with people. Telling my family members the things to learn…’ (FGB2:P18, male, aged 21)

‘Starting from the three days of training it decreased the level of stress because now I was able to call at home [*family in the rural area*] …and tell them they can use these things [*the vitamins the participants heard about*] …’ (FGB2:P16, male, aged 22)

#### Sub-theme 3.2: Developing adaptive coping strategies

The participants reported positive adaptive coping strategies in their daily lives to protect themselves and their families, demonstrating a sense of self-agency. The following direct quotations support the participants’ experience of adaptive coping:

‘I became a lot more strict with handwashing properly…a lot more cautious about wearing PPE and where I wore it and wearing the right stuff…social distancing…didn’t appreciate when people got into my space…made me a lot more aware.’ (FGA1:P3, female, aged 22)

‘I’m getting better as days goes and when I can have a group chat where I talk about ok, this is how I feel and …this is what is happening in my ward and this is how my superiors do things like in response to the COVID-19 pandemic.’ (FGA1:P1, male, aged 25)

A further positive aspect that was reassuring for them was that of the participants supporting one another while they were separated from their families. The following direct quotations support the participants’ experience of supporting each other:

‘…[*M*]ost of us were sharing that on WhatsApp and Facebook. It didn’t only help us but also helped our friends, family members, and colleagues. It also made us very close because we started checking each other and updating each other on what was happening each day…’ (FGB2:P20, male, aged 27)

‘It was beneficial knowing I could message someone and they would be able to support and understand me…nice that [*I*] had other students [*on WhatsApp*] because regardless of where you do your placements, the same challenges are prevalent throughout…’ (FGA2:P6, female, aged 29)

## Discussion

During the pandemic, nursing students’ return to the clinical setting to complete the clinical requirements of their degrees presented them with a new unknown scenario, filled with uncertainties and fears influenced by the environment and their different contexts. The discussion follows the themes identified in the results of the study.

### Primary appraisal of the COVID-19 pandemic

The participants’ initial appraisal of returning to clinical practice during the pandemic reflected the global analogies of the clinical setting as the ‘frontline’, linked stress and ‘Coronavirus Anxiety’ (Lee 2020). In this study, the participants’ initial appraisal of vulnerability arose in a different context to international students who were called to ‘opt-in and help’ (Monforte-Royo & Fuster 2020; Townsend 2020), as students were given the option to defer but were returning to complete their degrees.

The participants reported that they anticipated that the clinical setting was now a changed, unknown environment and they were uncertain as to what to expect. These perceptions resulted in anticipation of their return perceived as stressful, filled with anxiety and fear of COVID-19, also described by Lee (2020). Moreover, and confirming studies with similar students in China and the United Kingdom (Cao et al. 2020; Swift et al. 2020), students reported anxiety linked to delays in academic activities. However, in this study, the primary stressor was the return to the COVID-19 clinical setting.

A key fear and source of anxiety was the safety and protection of themselves and their families. Some concerns regarding their safety were linked to fears around PPE acquisition, with media reports on the shortage of access (Maben & Bridges 2020) and supply chains challenges (McMahon et al. 2020). A second major fear was about infecting their family, not unlike the South African HSRC (2020) study (*n* = 7607), where 46.4% of the health worker respondents were significantly concerned (*p* < 0.001) about transmitting the virus to family members, with a higher proportion of nurses (39.4%) compared to the medical practitioners (29.3%) ‘highly concerned’.

The family also had a supportive role and influence, and the family structure provided a protective environment for students. Where a family was perceived to be supportive, students’ coping resources were heightened, facilitating adaptive coping (Lazarus & Folkman 1984). Similarly, Cao et al. (2020) reported that participants living with their parents experienced less anxiety. In South Africa, with its diverse cultures, the family is an important social entity as it nurtures members, in keeping with ‘Ubuntu’, an African philosophy of ‘oneness’ (Gade 2012).

### Contextual influence on primary appraisal

Geographic location before return to practice also influenced their initial perceptions. Participants described the perceptions of rural areas being safe, as opposed to the urban clinical practice areas. This may be due to both urban located universities being ‘hot spots’ at different periods during the pandemic (Daniel 2020). However, Cao et al. (2020) found that students in their study reported that living in urban areas was conducive to reducing anxiety related to COVID-19 pandemic, as urban areas were resource-intensive regarding material and education resources. Tang et al. (2020) confirmed this finding.

A third factor influencing the participants’ thoughts and feelings around the pandemic was the media’s portrayal of the pandemic. In the form of newspaper reports, news on TV and social media, the media has played a proactive role in perpetuating the discourse of fear in many people during the COVID-19 pandemic (Garfin, Silver & Holman 2020). From wearing masks, social distancing and daily statistics on active COVID-19 cases and death counts, the media has brought the pandemic to the forefront of most conversations and social interactions (Garfin et al. 2020). The Director-General of the World Health Organization warned against the dangers of an ‘infodemic’ that had escalated in the pandemic as people succumbed to misinformation leading to mistrust (The Lancet Infectious Diseases 2020). There was evidence that this has been associated with anxiety (Gao et al. 2020). Similarly, the HSRC (2020) South Africa health worker survey reported that the media heightened awareness and increased the perceptions of risk.

### Reappraisal to facilitate positive return to clinical practice

A theme that emerged was that the thoughts and fears experienced in anticipation did not remain constant as students returned to clinical practice. This was unlike the South African nurses surveyed in the HSRC study (2020), who reported the sense of being ill-equipped with the required knowledge and training in infection control measures. Participants in this study reported that as their knowledge about the pandemic increased, they made use of the opportunities that the clinical practice environment afforded them by eliciting peer support and educating their family and friends about the virus. This may be attributed to faculty’s work in orientation and preparation of the students bolstered by their resilience of working in clinical practice as senior students. This is consistent with the findings of Savitsky et al. (2020), who reported that resilient students use positive coping strategies in the face of adversity. Furthermore, the participants’ ability to access resources such as support from their facilitators, online information and exposure to working in clinical practice during the pandemic may have created a sense of camaraderie that they were not alone, contributing towards their coping and developing a sense of self-agency.

## Limitations

The use of online focus group discussions limited the development of field notes and participants’ engagement. The online setting is not an ideal setting to explore non-verbal communication cues that can add to the richness of the data. Furthermore, the online format might limit students’ willingness to volunteer information.

## Recommendations

Faculty preparing students to work in the clinical setting during a pandemic are to consider the students’ anticipatory perceptions and include a thorough orientation in terms of knowledge, infection control and dealing with stress in an ongoing support system.

## Implications

The implementation of supportive measures by faculty to students transitioning into situations of uncertainty, such as the pandemic, has a strong potential to contribute practice-ready and responsive neophytes to the nursing profession.

## Conclusion

In the nursing students’ transition from the perceived safety of their primary residence to returning to clinical practice during the pandemic, their anticipatory perceptions were of uncertainty and stress. Through preparation and the process of reappraisal, they were able to adapt and cope with the challenge of returning to clinical practice during the pandemic. This study provides a rich picture analysed through the prism of Lazarus and Folkman (1984) for students’ perceptions as they prepared to return to practice and the process of adaptation through knowledge and experience. It draws attention to faculty, and clinical practice managers recognising undergraduate students’ mental health needs and exercising their responsibility towards supporting them emotionally through demanding transitions, not limited to those experienced in the pandemic.
